# Prevention of Thromboembolic Events in Patients with COVID-19

**DOI:** 10.1055/a-1576-6201

**Published:** 2021-08-02

**Authors:** Surbhi Warrior, Elizabeth Behrens, Joshua Thomas, Sefer Gezer, Parameswaran Venugopal, Shivi Jain

**Affiliations:** 1 Department of Internal Medicine, Rush University Medical Center, Chicago, Illinois, United States; 2 Division of Hematology-Oncology, Rush University Cancer Center, Chicago, Illinois, United States

The novel coronavirus disease 2019 (COVID-19)-associated coagulopathy is a known cause of significant morbidity and mortality in patients affected by coronavirus. The proposed pathogenesis of hypercoagulability in COVID-19 patients is attributed to Virchow's Triad, wherein hypoxia, severe inflammation, and cytokine storm cause endothelial injury, activation of the coagulation cascade, and immobilization in critically ill patients, all contribute to increased risk of thrombosis. 1 Patients requiring intensive care unit (ICU) admission have increased risk of thrombosis despite standard dose of thromboprophylaxis. 2 3 4 COVID-19 treatment has focused on targeting the unregulated inflammatory state to decrease incidence of COVID-19-related complications, including thrombosis.

Due to increased risk of thromboembolism, prophylactic anticoagulation is recommended in all hospitalized COVID-19 patients and intermediate to therapeutic dosing is suggested in patients with severe COVID-19, such as those requiring ICU admission. 5 6 In addition to hypercoagulability, COVID-19 patients are also at an increased risk for bleeding events due to variance in platelet production and destruction, consumption of coagulation factors in the setting of severe inflammation, increased exposure to and dosing of anticoagulation, and requirement of renal replacement therapy (RRT), indicating the need for further investigation into the long-term effect of escalated thromboprophylaxis dosing in this patient population. 7 8

This analysis was done to better understand the prevention of thrombosis in hospitalized COVID-19 patients by way of analyzing risk factors, impact of anticoagulation, and how COVID-19-specific therapies played a role in incidence of thrombosis and mortality. Adult patients with COVID-19 between March 1, 2020, and June 26, 2020, hospitalized at our institution were included in this retrospective analysis. Thrombotic events, including deep vein thrombosis (DVT), pulmonary embolism (PE), and stroke, were assessed by imaging such as leg compression ultrasounds, computed tomography (CT) pulmonary angiography, and head CT or magnetic resonance imaging (MRI), respectively, for patients who had signs and symptoms of venous thromboembolism (VTE) during hospitalization for COVID-19 to confirm the presence of thromboembolism.

Out of a total of 1,265 COVID-19-positive hospitalized patients, there were 323 patients with signs and symptoms for thromboembolism, defined by getting a CT head or MRI, CT pulmonary angiography, or leg compression ultrasounds. Of all COVID-19-positive hospitalized patients, 138 (10.9%) patients had a confirmed thromboembolism with characteristics shown in Table 1 . Subgroup analysis on incidence of thromboses in all COVID-19 patients showed no difference between gender (male, 11.6% and female, 9.9%; *p* = 0.3375), race and ethnicity (African American, 11.8%; Hispanic, 9.4%; other, 18.4%; White, 9.4%; *p* = 0.0899), or body mass index (BMI; normal defined as <25 kg/m ^2^ at 8.9%, overweight defined as 25–30 kg/m ^2^ at 11.17%, obese defined as >30 kg/m ^2^ at 12.6%; *p* = 0.2183). The average age of COVID-19 patients with thrombosis was significantly greater than the average age of COVID-19 patients without thrombosis, 59.6 years versus 57.2 years ( *p* = 0.0005). Hispanic patients with thrombosis had a significantly higher mortality rate of 51% compared with mortality rate of other groups with thrombosis: African American, 18%; other, 29%; or White, 20% ( *p* = 0.0020).

**Table 1:** Patient characteristics of COVID-19 patients with thrombosis

Characteristics	Thrombosis *n* = 138 *n* (%)
Age (y) Mean (range)	59.6 (23–89)
Young <45	18 (13.0)
Middle aged (45–65)	78 (56.5)
Elderly >65	42 (30.4)
Sex	
Male	82 (59.4)
Female	56 (40.5)
Race and ethnicity	
White	15 (10.9)
African American	56 (40.5)
Hispanic	51 (36.8)
Other	16 (11.6)
BMI (kg/m ^2^ )	
Normal (<24.9)	36 (26.1)
Overweight (25–29.9)	41 (29.7)
Obese (>30)	56 (40.5)
Unknown	5 (3.6)
Thromboembolism type a	
Deep vein thrombosis	71 (51.4)
Pulmonary embolism	61 (44.2)
Stroke	26 (18.8)
Anticoagulation prior to thrombosis	
None	30 (21.7)
Prophylactic	66 (47.8)
Enoxaparin	43 (31.2)
Heparin	23 (16.7)
Therapeutic	41 (29.7)
Enoxaparin	29 (21.0)
Heparin	5 (3.6)
Apixaban	4 (2.9)
Bivalirudin	3 (2.2)
Unknown	1 (0.7)
COVID-19-related therapy b	
Steroids	64 (46.4)
Tocilizumab	36 (26.1)
Remdesivir	18 (13.0)
Hydrocychloroquine	48 (34.8)
Mortality	44 (31.9)
Hospital characteristics	
Length of stay Mean (range)	20.5 (1–92)
ICU admission	116 (84.1)
ICU length of stay Mean (range)	18.7 (0.08–91.2)
Mechanical ventilation	98 (71)
D-dimer > 6	68 (49.2)
Renal replacement therapy	45 (32.6)

Abbreviations: BMI, body mass index; COVID-19, novel coronavirus disease 2019; ICU, intensive care unit;

a Some patients had multiple types of thromboembolism.

b Some patients received multiple types of COVID-19 therapy.

Incidence of thrombosis in ICU patients with COVID-19 was significantly greater than in non-ICU patients with COVID-19, 23.9 versus 2.95% ( *p* < 0.0001). COVID-19 patients who had thrombosis had significantly higher need for intubation and mechanical ventilation compared with COVID-19 patients who did not have thrombosis, 71.0 versus 24.8% ( *p* = 0.00001). Incidence of thrombosis in COVID-19 patients with D-dimer levels >6x ULN was significantly greater than COVID-19 patients who had lower D-dimer levels, 36.6 versus 8.2% ( *p* < 0.0001). The mortality rate of patients with thrombosis who received RRT was significantly higher than those who did not require RRT, 47.4 versus 26.0% ( *p* = 0.0161). Mortality rate in COVID-19 patients with thrombosis was significantly higher than COVID-19 patients without thrombosis, 31.9 versus 10% ( *p* < 0.0001). The average hospital length of stay (LOS) in patients with thrombosis was significantly higher than hospital LOS in patients without thrombosis, 20.6 versus 9.8 days ( *p* < 0.0001).

Among the COVID-19-positive patients who had thrombosis, we studied the impact of COVID-19 directed therapy on the incidence of thrombosis. Analysis showed that the incidence of thrombosis in COVID-19 patients who received steroids was significantly less at 14% as compared with incidence of thrombosis in other COVID-19 treatment: tocilizumab, 25% ( *p* = 0.0031); hydroxychloroquine, 42% ( *p* < 0.0001); and remdesivir, 72% ( *p* < 0.0001). A Cox's regression model showed that when adjusting for age, race, ethnicity, and BMI for demographics and ICU admission for severity of disease, there was no correlation between patients who received steroids compared with those who did not receive steroids to incidence of thrombosis (hazard ratio [HR] = 0.959, *p* = 0.8452).

Adjusting for demographics, as well as ICU admission status to account for severity of disease, a Cox's regression model showed that there was no difference in mortality between COVID-19 patients who received therapeutic versus prophylactic anticoagulation prior to diagnosis of thrombosis (HR = 1.0538, *p* = 0.8831). The bleeding rate in COVID-19 patients with thrombosis was significantly higher than reported bleeding rates for hospitalized nonCOVID-19 patients on anticoagulants, 12.3 versus 7.2% ( *p* < 0.05). 5 9 The majority of bleeding events occurred after a noted thrombosis (58.8%) on therapeutic dosing of anticoagulation (64.7%), in the ICU (70.6%), and were World health Organization (WHO) grade 4 (58.8%) as seen in Table 2 . Only 17.6% of COVID-19 patients with thrombosis who had bleeding had thrombocytopenia defined as platelet count <100 × 10 ^3^ µL at the time of bleeding.

**Table 2:** Bleeding events in COVID-19 patients with thrombosis

Gender	Age (y)	Type(s) of bleed	WHO bleeding grade	Platelets at bleed (K/µL)	Bleed before or after thrombosis	AC before bleed	AC after bleed	ICU at the time of bleed	Fatal bleed	Hospital mortality	Comments
M	66	Hemorrhagic stroke	Grade 4	265	Same time	Prophylactic (UFH)	Therapeutic (UFH)	Yes	No	Alive	Transformation from embolic stroke
M	62	Hemithorax	Grade 4	55	After	Therapeutic (argatroban)	Therapeutic (bivalirudin)	Yes	No	Alive	ECMO
M	47	Nasal, oropharngeal	Grade 4	143	After	Therapeutic (argatroban)	Therapeutic (argatroban)	Yes	No	Alive	ECMO
M	55	Hemorrhagic stroke	Grade 4	240	After	Therapeutic (LMWH)	Therapeutic (LMWH)	Yes	No	Deceased	
F	44	Nasal, oropharngeal, rectal, vaginal	Grade 4	264	After	Therapeutic (LMWH)	None	Yes	No	Alive	Recurrent Bleed
M	46	Nasal and hemorrhagic stroke	Grade 4	154	After	Therapeutic (LMWH), tPA	None	Yes	No	Deceased	
F	58	Hemoptysis	Grade 2	13	After	None	None	No	No	Alive	Current AML
F	45	Nasal	Grade 2	102	After	Therapeutic (bivalirudin)	Therapeutic (bivalirudin)	Yes	No	Alive	ECMO
M	48	HD catheter bleed	Grade 3	147	After	Prophylactic (UFH)	None	Yes	No	Deceased	
M	77	Oropharyngeal and hemorrhagic stroke	Grade 4	117	Same time	Therapeutic (LMWH)	Therapeutic (LMWH)	No	No	Alive	DIC on admission
M	72	Nasal	Grade 2	322	Before	None	Prophylactic (LMWH) and tPA	No	No	Deceased	
F	63	Hemorrhagic stroke	Grade 4	186	After	Therapeutic (LMWH)	Therapeutic (bivalirudin)	No	No	Alive	
M	45	Oropharyngeal	Grade 2	51	Before	Therapeutic (LMWH)	Prophylactic (LMWH)	Yes	No	Deceased	
M	32	GI	Grade 2	288	After	Therapeutic (LMWH), warfarin	Therapeutic (UFH)	Yes	No	Alive	
F	60	GI	Grade 2	308	Before	Prophylactic (LMWH)	Therapeutic (apixaban)	Yes	No	Alive	
M	51	Hemorrhagic stroke	Grade 4	224	Same time	Therapeutic (LMWH)	None	Yes	No	Deceased	
F	88	Hemorrhagic stroke	Grade 4	167	Same time	None	None	No	No	Alive	Discharge to hospice

Abbreviations: AC, anticoagulation; COVID-19, novel coronavirus disease 2019; DIC, disseminated intravascular coagulation; ECMO, extracorporeal membrane oxygenation; F, female; GI, gastrointestinal; ICU, intensive care unit; LMWH, low molecular weight heparin; M, male; UFH, unfractionated heparin; WHO, World Health Organization; AML, acute myeloid leukaemia; HO, hemodialysis; TPA, tissue plasminogen activator.

Our study showed increased incidence of thrombosis in hospitalized COVID-19-positive patients. These patients also had longer hospital LOS and increased mortality. Risk factors identified for thrombosis include older age and Hispanic ethnicity. Analysis of hospital course showed patients with D-dimer > 6x ULN, ICU admission, RRT requirement, and mechanical ventilation had higher incidence of thrombosis and higher mortality. A risk stratification system based on these factors to identify COVID-19-positive patients who are at high risk for thrombosis may be beneficial for early identification and intervention to reduce hypercoagulability-associated mortality rate in hospitalized COVID-19 patients.

We also found that the incidence of thrombosis was significantly less in patients who received any kind of steroids which was not the case with other COVID-19 therapies. When adjusting for demographics and severity of disease, this was not statistically significant. This could be because this study looked at patients in the first round of the pandemic where only severely sick ICU patients were receiving COVID-19-related therapies and because there were differences in steroid doses given among patients. The RECOVERY trial demonstrated reduced mortality in COVID-19 patients who received dexamethasone, and our data show decreased incidence of thrombosis with steroids and how thromboembolism is associated with increased mortality rates. 10 This may suggest that steroids may help reduce mortality by diminishing the proinflammatory state that leads to hypercoagulability. Steroids have been previously shown to increase risk of thrombotic events in non-COVID-19 patients, but the pathogenesis of thrombosis in COVID-19 is starkly different than in other disease states. Prospective studies need to be done with standard dosing of steroids in all COVID-19 patients to understand if steroids should be used in COVID-19 patients with high risk for thrombosis.

There was no difference in mortality in COVID-19 patients who had prophylactic versus therapeutic dosing of anticoagulation, even when adjusting for demographics and severity of disease. Our study specifically looked at empiric prophylactic and therapeutic anticoagulation given to COVID-19 patients prior to a thrombotic event to assess mortality benefit. Our study supports society guidelines that prophylactic anticoagulation should be given to all hospitalized COVID-19-positive patients in the absence of confirmed or high suspicion for VTE, but prospective studies need to be done to evaluate if there is further benefit for increased doses of thromboprophylaxis in this high-risk population. 6

The majority of bleeding events were severe (WHO grade 4) and occurred in the ICU, where patients were thought to be at highest risk for thrombosis and were empirically treated with therapeutic anticoagulation. Given that most patients had normal platelet counts, this bleeding was not attributed to disseminated intravascular coagulation (DIC) which has been commonly noted in COVID-19 patients. 8 Risk factors, such as stress gastritis, contributed to bleeding in critically ill patients, and long-term morbidity of bleeding events should be taken into consideration. We suggest that there should be careful monitoring for signs and symptoms of bleeding in critically ill COVID-19 patients, while they are on therapeutic anticoagulation.

Our findings support the significant impact of coagulopathy in COVID-19 patients which leads to longer hospital LOS, morbidity, and mortality. There is much work that needs to be done in understanding which patients would most benefit from intermediate to therapeutic dose of anticoagulation while taking into consideration high bleeding rates in this population. Our study also shows that steroids may play a role in reducing thromboembolism in these patients which has not previously been studied. As COVID-19 continues to affect people worldwide, an emphasis needs to be placed on better understanding how to prevent COVID-19-related coagulopathy. Results from randomized control trials are eagerly awaited to provide further guidance in this area.

## Notes

**What Is Known about This Topic?**
There is an increased risk of thrombosis in the novel coronavirus disease 2019 (COVID-19) patients.

- COVID-19 leads to a hypercoagulable state due to increased inflammatory response. Anticoagulation is recommended to decrease risk of thrombosis in COVID-19 patients.
- There are conflicting recommendations whether COVID-19 patients should be on prophylactic versus intermediate versus therapeutic dosing of anticoagulation.
- Low molecular weight heparin is the anticoagulant of choice for COVID-19 patients.

**What Does This Paper Add?**
What groups of patients are at highest risk for thrombosis and thrombosis-associated mortality?

- Hispanic patients and older age patients are at higher risk for thrombosis and the novel coronavirus disease 2019 (COVID-19) mortality.
- Patients with D-dimer > 6x ULN, intensive care unit (ICU) admission, renal replacement therapy (RRT) requirement are more likely to have thrombosis.
- These high risk groups should be closely monitored for signs and symptoms of thrombosis.
  Is there any COVID-19 related treatment to reduce risk for thrombosis?
- Patients who received steroids had lower incidence of thrombosis
  Should COVID-19 patients get prophylactic versus therapeutic anticoagulation?
- There was no difference in mortality in COVID-19 hospitalized patients who got prophylactic versus therapeutic anticoagulation before thrombosis.
- There were high bleeding rates in COVID-19 patients showing that escalation of anticoagulation does not come without risks.
